# A Medication Synchronization Program and Blood Pressure Levels in a Community Pharmacy: Protocol

**DOI:** 10.2196/12527

**Published:** 2019-04-01

**Authors:** Anthony Pattin, Rebekah L Panak, Rebecca Hunold, Abagail Kirwen, Samantha R Minnich, Tian Chen

**Affiliations:** 1 Department of Pharmacy Practice College of Pharmacy and Pharmaceutical Sciences The University of Toledo Toledo, OH United States; 2 Department of Mathematics and Statistics College of Natural Science and Mathematics The University of Toledo Toledo, OH United States

**Keywords:** pharmacy practice, medication synchronization, hypertension

## Abstract

**Background:**

The lack of adherence to prescribed antihypertensive medication occurs in 50% of patients and leads to poor health outcomes and increased medical costs. Consistent use of antihypertensive medications among patients with hypertension is essential to the reduction of short- and long-term cardiovascular complications. Strategies to improve medication adherence include syncing prescription medications in the pharmacy, which allow patients to retrieve chronically prescribed medications in one visit. The adoption of medication synchronization has been shown to improve adherence to medications; however, there is a lack of data showing if the intervention reduces blood pressure and improves long-term health outcomes.

**Objective:**

This study aims to determine the association between participation in an appointment-based medication synchronization service and blood pressure levels among patients on antihypertensive medications.

**Methods:**

This longitudinal prospective cohort study will observe changes in blood pressure among individuals in a medication synchronization program and those in a usual care group. Patients on at least two antihypertensive medications and four total medications have been recruited to participate in the study. All participants will be required to have at least a 6-month history of filling prescriptions at the pharmacy prior to enrollment in the study. Based on an estimated standard deviation of 14 mmHg, a sample size of 70 participants provides approximately 80% power with a two-sided .05 significance to detect a difference of 9 mmHg blood pressure between the two cohorts.

**Results:**

As of the publication of this paper, patients are completing final blood pressure visits at the pharmacy and medication data are being collected from the pharmacy. Once patients complete all blood pressure visits, data analysis will begin.

**Conclusions:**

This study will link medication synchronization and changes in blood pressure levels among individuals with hypertension. This study will provide preliminary data for a randomized clinical trial that will assess the impact of medication synchronization on blood pressure.

**International Registered Report Identifier (IRRID):**

DERR1-10.2196/12527

## Introduction

### Background

Despite the multitude of treatment options for the management of hypertension, heart disease and stroke remain the leading causes of death attributed in part to high blood pressure. Among the 75 million Americans with hypertension, 74.9% of patients are being treated and only 52.5% of patients are under control [1]. Although effective medications to lower blood pressure have been around for decades, lack of medication adherence occurs among 50% of patients and leads to poor health outcomes [2]. Medication nonadherence is attributed to multiple factors, including lack of health system coordination, asymptomatic disease, patient-specific factors, and complexity of drug regimens [3].

Pharmacy-based strategies to improve medication adherence include the incorporation of synchronization, which enables patients to retrieve multiple medications systematically on one predetermined date [4-6]. Currently, there is limited literature suggesting medication synchronization might improve health outcomes in chronic conditions such as hypertension. One study examined the effect of medication synchronization or an education program on hypertensive health outcomes of patients in a community pharmacy setting [7]. After 4 months of enrollment in medication synchronization, patients achieved an average systolic blood pressure (SBP) reduction of 4 mmHg (*P*=.04) [7]. However, it is important to note that the control and education groups both achieved significant reductions in SBP (9 mmHg, *P*=.001 and 10 mmHg, *P*<.001, respectively) [7]. These significant findings may have been due to the feedback patients received, as all groups had their blood pressure taken and were educated when picking up their medications [7]. A review of participants recruited for the study indicated that baseline blood pressures were low at the start of the study—average baseline SBP of 138 mmHg and diastolic blood pressure (DBP) of 79 mmHg—which may have contributed to a lack of change at the conclusion of the study [7]. Additionally, there was no change in the patients’ reported self-adherence to medications within any groups [7]. This may suggest that ensuring patients have their medications by implementing medication synchronization alone may not be enough to improve patient adherence and, in turn, health outcomes.

Other literature may not specify medication synchronization as the intervention used. Although other studies may not be identically comparable to medication synchronization, there are some similarities to the interventions performed that merit investigation. One such example is the Federal Study of Adherence to Medications in the Elderly (FAME) study, which examined the effect of a pharmacy care program on patient adherence and persistence [8]. This study also examined the pharmaceutical care program’s effect on blood pressure and cholesterol outcomes. The authors found that improved adherence was associated with improvements in SBP, with the pharmaceutical care group achieving greater SBP reduction than usual care (-6.9 mmHg and -1.0 mmHg, respectively, *P*=.04) [8]. The pharmaceutical program consisted of providing medication education, dispensing of medications using blister packaging, and regular follow-up with a pharmacist every 2 months. Individualized education was provided to teach patients about drug names, indications, strengths, adverse effects, and instructions for use during visits [8]. Medication education is commonly performed by pharmacists and can be a component of the medication synchronization intervention along with regular follow-up. Medication synchronization requires pharmacists to go over the medication list with the patient each month. In doing so, regular follow-up occurs and the patient or pharmacist may have questions that lead to medication education or more comprehensive medication therapy management services.

Though there are similarities between medication synchronization and the FAME study intervention, the key difference is the use of blister packaging in the study. Thus, the study cannot directly be compared to a medication synchronization intervention due to the inclusion of compliance packaging. Although medication synchronization has been shown to indirectly improve medication adherence, it is not known if utilization leads to reduced blood pressure. Therefore, we aim to determine the association between participation in a medication synchronization service and blood pressure levels among patients on antihypertensive medications.

The overall objective of this project is to determine the association between participation in an appointment-based medication synchronization service and blood pressure levels among patients on antihypertensive medications. Other study objectives are to examine whether the effect of an appointment-based medication synchronization service on patients’ blood pressure vary based on time-invariant demographic characteristics and time-variant characteristics of the subjects. Data generated from this study will determine the appropriate study size for a larger clinical trial of the intervention.

### Aims and Hypothesis

Aim 1 is to examine the association between enrollment in an appointment-based medication synchronization program and study participants’ blood pressure levels. Hypothesis 1 is as follows: consistent enrollment within a medication synchronization service will be associated with lower blood pressure measurements. This hypothesis will be tested using blood pressure ascertained over a follow-up period of up to 10 months.

Aim 2a is to examine whether the effect of an appointment-based medication synchronization program on patients’ blood pressure varies based on time-invariant demographic characteristics of patients, such as gender, age, education, income, and ethnic origin.

Aim 2b is to examine whether the effect of an appointment-based medication synchronization program on patients’ blood pressure varies based on time-variant characteristics of patients, such as weight, lifestyle change, adverse health behavior, ethanol use, frequency and intensity of exercise, adverse drug reaction, the number of medications, and adherence rates.

Aim 3 is to describe the level of patient satisfaction with enrollment in an appointment-based medication synchronization program.

## Methods

### Study Design

This is a longitudinal prospective study to observe whether enrollment in an appointment-based medication synchronization program will change SBP and DBP levels in patients with hypertension (ie, SBP/DBP of 140/90 mmHg or more) [9]. Two cohorts of patients will be recruited from pharmacy patrons who will volunteer to enroll in the study.

Group 1 (medication synchronization group) comprises patients voluntarily participating in the study and who will enroll in the appointment-based medication synchronization program.

Group 2 (traditional pharmacy medication pick-up group) comprises patients voluntarily participating in the study and who refuse to participate in the appointment-based medication synchronization program.

### Human Subjects

All procedures, informed consent protocols, and study documents were approved by the University of Toledo Biomedical Institutional Review Board. Study procedures were all approved by the pharmacy practice site.

### Informed Consent

All research subjects will sign written informed consent forms prior to participating in the study.

### Study Population

**Figure 1:**
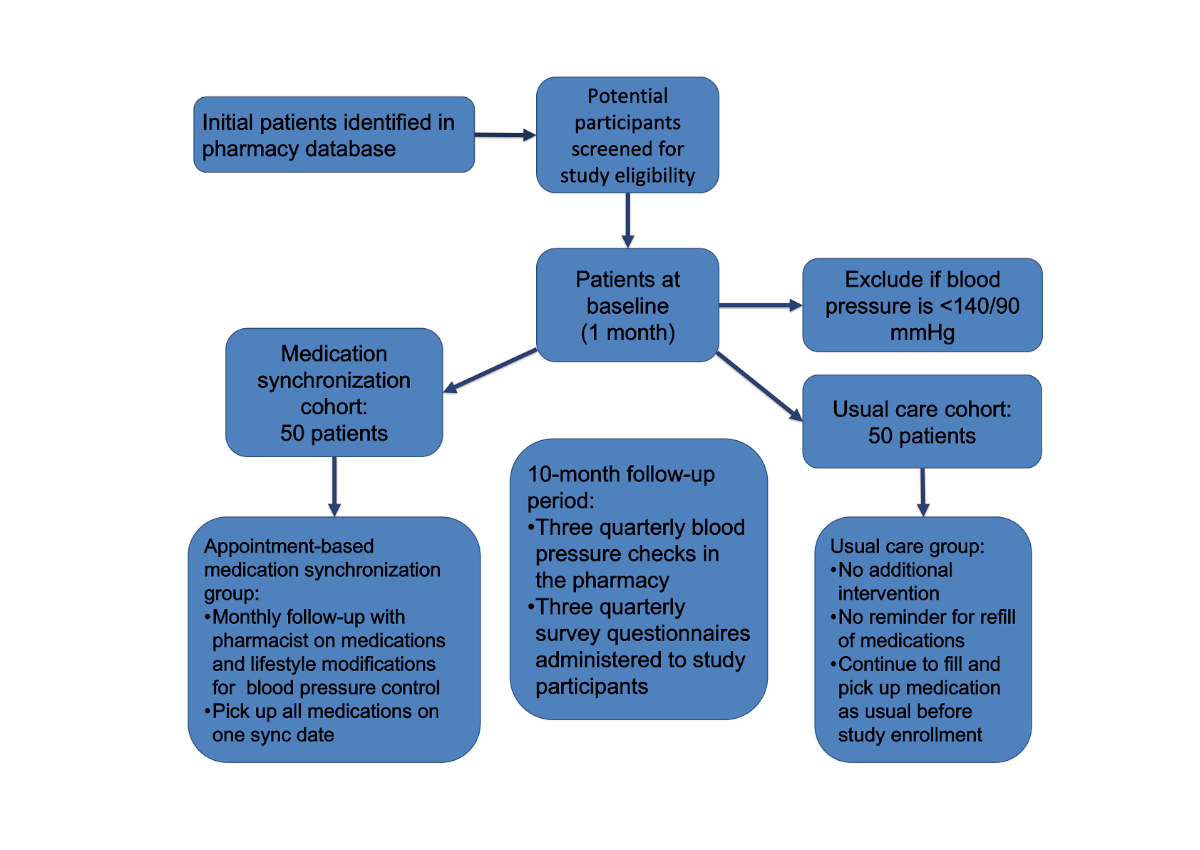
Study diagram.

Recruitment will occur among patients that utilize an independent pharmacy for prescription services in Toledo, Ohio. The pharmacy’s computer software will be screened for patients that potentially meet the inclusion criteria (see Figure 1). Up to 100 patients eligible to participate based on inclusion and exclusion criteria will be enrolled in the study. Textbox 1 describes inclusion and exclusion criteria. A flyer describing the study will be posted in the pharmacy to recruit individuals. The flyer will emphasize that participation is voluntary and that a participant may withdraw at any time during the study. The flyer encourages potential participants to call the principal investigator or a coinvestigator if they are interested in learning more about the study. A script was developed that describes the nature of the phone call. Patients interested in the study will be asked to visit the pharmacy to meet with a study investigator who will provide more information about the study. Risks and benefits of the study will be discussed with the patient. If, after meeting in person, the individual is interested in participating in the study, they will be invited to complete the informed consent document. Subjects may withdraw from the study at any time without prejudice to their care.

### Study Procedures

After the baseline clinic visit, patients that meet the study inclusion and exclusion provisions will be followed by cohort depending on the services they decide to utilize in the pharmacy. Participants will be categorized as participating in medication synchronization or traditional medication pick-up group. Participation in any of these pharmacy services are voluntary and are a part of care offered by the pharmacy. Participants will notify investigators as to which cohort they would like to participate in at the month-1 research visit.

The baseline visit procedure includes confirmation that all inclusion and exclusion criteria are satisfied, verification of participant consent and contact information, and completion of baseline data survey, including obtaining blood pressure for analysis. The follow-up visit schedules for data collection do not differ by cohort groups. Table 1 provides more detail about scheduled research visits and information that will be collected. For data collection in both groups, all participants will have visits at months 1, 4, 7, and 10. For event ascertainment, participants in both groups will be queried regarding the occurrence of a possible event on the same schedule, specifically every 3 months. Follow-up in-person visits at the pharmacy will involve gathering of blood pressure data. Follow-up phone call surveys will involve gathering data on changes in health status and satisfaction with pharmacy services.

A multitude of physical measures and medication data will be collected throughout the study via five research pharmacy visits or four phone call surveys. A one-time medical history will be collected at baseline to serve as a screening tool as well as an eligibility and stratification factor. The following physical measures will be collected at baseline: seated blood pressure, weight, height, gender, age, medical history, sociodemographic data, and smoking and alcohol usage. Seated blood pressure, physical measures, and questionnaires will be gathered during pharmacy visits, while all other information, including health status and satisfaction with pharmacy services, will be collected via phone call surveys. Sociodemographic information will reflect age, race, date of diagnosis, ethnicity, gender, level of education, marital status, persons living with participants, and US postal code. This data will be used to identify eligible participants and to characterize the final study population. Baseline medication data include the number of medications, type of medications, and adherence. *Proportion of days covered* will be utilized to assess medication adherence. Monthly medication data will be collected to evaluate prescription records to assess adherence. Seated blood pressure, weight, sociodemographic data, and smoking and alcohol usage will be collected every 3 months starting from the first month of the study.

**List of inclusion and exclusion criteria for study.**

Inclusion criteria:

- Subject is an adult, 18 years of age or older.
- Subject is able to read, understand, and sign a written informed consent form to participate in the study.
- Subject has systolic blood pressure >140 mmHg or diastolic blood pressure >90 mmHg during the baseline period.
- Subject has been an established patron of Toledo Family Pharmacy Inc for at least six previous refills of blood pressure medications.
- Subject has filled more than four prescriptions at Toledo Family Pharmacy each month for the past 3 months.
- Subject has a history of hypertension and has been prescribed at least two antihypertensive medications.
- Subject possesses an active phone number where they can be reached.
- Subject is willing and able to comply with the study protocol.

Exclusion criteria:

- Subject is participating in another clinical trial within four weeks of the baseline period involving an intervention that could affect the outcome measures of this study.
- Subject has end-stage renal disease and is on dialysis.
- Subject had a heart attack in the previous six months.
- Subject had a stroke in the previous six months.
- Subject is currently enrolled in an auto-delivery program or medication packaging program.
- Subject has a psychiatric or mental disorder that, in the judgment of the investigator, could interfere with provision of informed consent or completion of survey questionnaires.
- Patient has a designated power-of-attorney that signs for medications, which renders them not competent to sign the informed consent form.

**Table 1:** Study timeline and data collection.

Data to be collected		Baseline	Month									
			1	2	3	4	5	6	7	8	9	10
**Physical measures**												
	Seated blood pressure	x	x			x			x			x
	Weight	x	x			x			x			x
	Height	x										
**Questionnaires**												
	Gender	x										
	Age	x										
	Medical history	x										
	Sociodemographic data	x	x			x			x			x
	Smoking and alcohol use	x	x			x			x			x
**Medication data**												
	Number of medications	x	x	x	x	x	x	x	x	x	x	x
	Cost of medications	x	x	x	x	x	x	x	x	x	x	x
	Type of medications	x	x	x	x	x	x	x	x	x	x	x
	Adherence	x	x	x	x	x	x	x	x	x	x	x

The pharmacy will supply the research team with medication data on each of the participants enrolled in the study each month. Data provided will include the number of medications, names of medications, costs of medications, the date when the prescription was filled, and the date when the prescription was picked up from the pharmacy. For each drug, the quantity filled, days’ supply, and prescription number will be obtained. Protected health information collected in the study will be maintained on a key code sheet. This code sheet will be locked in the principal investigator’s office at the University of Toledo. A code will be on all data collection forms to refer to each participant in the study. These efforts will maintain research participants’ confidentiality throughout the study. Data collected on data forms will be maintained in a locked cabinet in the principal investigator’s office, separate from the key code sheet. Data collected on code sheets will also be transcribed to electronic format using REDCap provided by the University of Toledo.

### Data Analysis

By assuming a medium effect size of 0.75 times standard deviation for the intervention and set power at 0.8 with a two-sided alpha of .05, the sample size needed is 70 participants, with 35 in each group, allowing for up to 20% attrition. Based on the estimated standard deviation of 14 mmHg, the proposed sample size will provide approximately 80% power to detect a difference as small as 9.5 mmHg between intervention and control groups [7]. Descriptive statistics will be presented as mean (SD) for continuous data and percent frequency (interquartile range) for categorical data to describe patients’ characteristics.

Given the observational nature of the study and patients’ self-selection of the treatment arms, inverse-probability -of-treatment weighted (IPTW) estimation of marginal structural models (MSMs) will be employed in this study. This estimation addresses both sources of potential bias, such as confounders or covariates (eg, age, income, and medication profiles) and missing data under the missing-at-random (MAR) assumption, the most popular informative mechanism in practice. MSMs are a class of statistical models used for causal inference in epidemiology and use a multi-step estimation strategy to control for the effect of confounding variables, allowing the investigator to obtain unbiased estimates [10-12]. Among the several methods that have been proposed to estimate the parameters in MSMs, IPTW is the most commonly used one to address confounding variables [13]. It tries to control for confounding variables by assigning each observation a weight and uses this to create a *pseudo-population,* where treatment arm and control arm are balanced over the confounder [10]. When applied to our study, an MSM consists of three submodules that models the following: (1) the probability of receiving treatment (ie, synchronized arm) for each participant and the weights of the IPTW estimator are simply the inverse of those probabilities; (2) the probability of missing data under MAR and the inverse of those probabilities are used as weights to account for missing values; and (3) the effects of synchronization on blood pressure using reweighted samples. Logit link will be used in both auxiliary modules for the treatment arm and missing data. By controlling for observed confounders and missing data mechanisms through the two auxiliary modules, the main module will provide valid inference about treatment differences in the absence of hidden bias. Statistical significance is set at an alpha of .05 for all analyses.

## Results

Funding was provided on May 12, 2017. As of the publication of this paper, patients are being enrolled in the study and completing follow-up visits in the pharmacy. Also, as of the publication of this paper, investigators are collecting prescription data provided by the pharmacy for all participants in the study. Data collection and analysis will be completed by May 2019.

The medication synchronization service will be delivered as described in the American Pharmacists Association Foundation’s *Pharmacy’s Appointment-Based Model Implementation Guide* *for Pharmacy Practices* [14]. There are three major components of medication synchronization, which include (1) prescription synchronization, (2) a monthly call to the patient, and (3) scheduled monthly appointments. For the prescription synchronization step, a patient should be assigned a sync day for all medications in the pharmacy. Additionally, the pharmacy may have to facilitate short or long fills, so medications can be synced for the assigned date.

Once a sync day has been assigned, a pharmacy staff member will call the patient at least a week before the sync date. This is to ensure there are no medication changes prior to filling medications and for the pharmacy staff to confirm if there have been any new medications added or medications that have been discontinued. Once the patient comes to pick up medications that have been synced, the pharmacist will briefly counsel the patient.

The monthly appointments are times when patients will come in to retrieve their prescriptions. This is also a time the pharmacist can provide some education to the patient on blood pressure management. This encounter is not meant to go beyond the usual patient consultation that occurs when a patient picks up a prescription. The intent of this consultation is to provide a focused means of providing patients with education on hypertension and medication use. A brief topic outline described in Table 2 has been developed to facilitate talking points for patients enrolled in the medication synchronization group of the study.

**Table 2:** Outline of counseling points for medication synchronization cohort.

Month	Pharmacist guide for counseling patients
1	Give American Heart Association sheet, *What is High Blood Pressure?* and discuss basic information about blood pressure: it is a silent disease.
2	Discuss multiple way to control blood pressure, including with diet and medications; discuss lifestyle.
3	Discuss treatment goals. What are my blood pressure targets?
4	How do my blood pressure medications work? Discuss each medication and how they each work.
5	Discuss my lifestyle and how it impacts blood pressure control. Is there one goal I can make to improve blood pressure control?
6	Discuss medication side effects. Review medications again and risks of side effects.
7	Discuss lifestyle and physical activity. What can I do to improve my fitness?
8	Discuss future goal to maintain or improve blood pressure levels.
9	Discuss future goal to maintain or improve blood pressure levels.
10	Discuss future goal to maintain or improve blood pressure levels.

## Discussion

Although there are studies that demonstrate medication synchronization has an impact on adherence metrics, there is still need for data to connect it with a clinical measure such as blood pressure. Data generated from this study will provide an association between medication synchronization and blood pressure levels. It will also determine effect size and the appropriate sample size for a future clinical trial of the intervention. Limitations of this study include lack of randomization at the point of entry in the study. Study investigators will use MSMs to overcome self-selection bias.

## Abbreviations

DBP: diastolic blood pressure
FAME: Federal Study of Adherence to Medications in the Elderly
IPTW: inverse-probability-of-treatment weighted
MAR: missing at random
MSM: marginal structural model
NACDS: National Association of Chain Drug Stores
SBP: systolic blood pressure
